# Involvement of Tricarboxylic Acid Cycle Metabolites in Kidney Diseases

**DOI:** 10.3390/biom11091259

**Published:** 2021-08-24

**Authors:** Alexis Paulina Jiménez-Uribe, Estefani Yaquelin Hernández-Cruz, Karla Jaqueline Ramírez-Magaña, José Pedraza-Chaverri

**Affiliations:** Departamento de Biología, Facultad de Química, Universidad Nacional Autónoma de México, Mexico City 04510, Mexico; estefani.hernandez@quimica.unam.mx (E.Y.H.-C.); karlaa_jaquelinee@comunidad.unam.mx (K.J.R.-M.); pedraza@unam.mx (J.P.-C.)

**Keywords:** mitochondria, TCA cycle metabolites, kidney diseases

## Abstract

Mitochondria are complex organelles that orchestrate several functions in the cell. The primary function recognized is energy production; however, other functions involve the communication with the rest of the cell through reactive oxygen species (ROS), calcium influx, mitochondrial DNA (mtDNA), adenosine triphosphate (ATP) levels, cytochrome c release, and also through tricarboxylic acid (TCA) metabolites. Kidney function highly depends on mitochondria; hence mitochondrial dysfunction is associated with kidney diseases. In addition to oxidative phosphorylation impairment, other mitochondrial abnormalities have been described in kidney diseases, such as induction of mitophagy, intrinsic pathway of apoptosis, and releasing molecules to communicate to the rest of the cell. The TCA cycle is a metabolic pathway whose primary function is to generate electrons to feed the electron transport system (ETS) to drives energy production. However, TCA cycle metabolites can also release from mitochondria or produced in the cytosol to exert different functions and modify cell behavior. Here we review the involvement of some of the functions of TCA metabolites in kidney diseases.

## 1. Introduction

Mitochondria are organelles that fulfill a wide variety of functions in the cell. In addition to being a bioenergetics node, they also serve as signal organelles that communicate to the rest of the cell through different mechanisms. For example, the production of reactive oxygen species (ROS) [1], calcium influx [2], adenosine triphosphate (ATP) levels that regulate adenosine monophosphate protein kinase (AMPK) activation [3], modulation of the immune response through mitochondrial DNA (mtDNA) [4], releasing of cytochrome c orchestrating apoptosis [5], and also through Krebs cycle metabolites [6]; that modulates cell adaptation to different conditions.

The tricarboxylic acid (TCA) cycle, also named the citric acid cycle and Krebs cycle (although this last name can be dissected in the three different cycles: the urea, the glyoxylate, and TCA cycles) was described by Hans Krebs and his colleagues [7]. It is known chiefly for producing electron donors, the reduced form of nicotinamide adenine dinucleotide (NADH), and the reduced form of flavin adenine dinucleotide (FADH_2_) to feed the electrons transport system (ETS). However, their intermediates also can serve as signal molecules to drive several cell functions.

TCA cycle metabolites were discovered as signal molecules mainly in cancer cells and were defined as oncometabolites that promote tumor progression [8]. However, recent evidence suggests that they are associated with diverse pathologies, including kidney diseases.

Kidneys are highly dependent on mitochondrial function due to their energy demand, particularly by the tubular nephron section, which exerts filtration and reabsorption functions. Hence, mitochondrial alterations, such as dynamics (fusion/fission), homeostasis (biogenesis/mitophagy), and bioenergetics, impact on kidney function.

In addition, other metabolic functions of the mitochondrial, such as the TCA cycle, could also be involved in kidney diseases.

## 2. A Brief Overview of the TCA Cycle

TCA cycle is an amphibolic pathway; the anabolic routes involve gluconeogenesis, transamination reactions, deamination reactions, and fatty acid synthesis; whereas catabolic routes oxidized components derived from carbohydrates, proteins, and lipids to generate electron donors and guanosine triphosphate (GTP) for energy production.

The cycle requires the condensation of acetyl coenzyme A (Acetyl-CoA) with oxaloacetate (OAA) to generate citrate; this reaction is catalyzed by the citrate synthase (CS). Citrate is then isomerized to cis-aconitate and further to isocitrate; aconitase activity is necessary for these reactions.

Isocitrate is dehydrogenated and decarboxylated to alpha-ketoglutarate (AKG) by the isocitrate dehydrogenase (IDH); in this reaction CO_2_ and NADH also are produced. AKG is further decarboxylated to succinyl CoA by the AKG dehydrogenase; in this reaction, as the previous, NADH and CO_2_ are produced. Succinyl CoA is converted to succinate by the action of succinyl CoA synthetase (also named succinate thiokinase); in this step, a molecule of GTP is produced. Succinate is dehydrated to fumarate by the succinate dehydrogenase (SDH), in this step, FADH_2_ also is produced. Fumarate is then hydrated to generate malate by fumarate hydratase (FH), also named fumarase. Finally, malate is dehydrogenated to generate OAA by malate dehydrogenase (MDH); in this reaction, the third molecule of NADH is also produced. The produced OAA could start the cycle again by condensing with Acetyl CoA [6,9].

**Figure 1:**
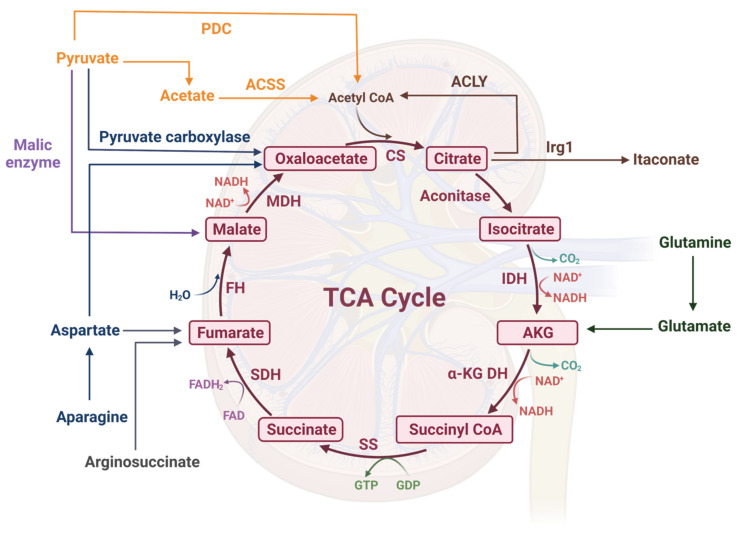
The tricarboxylic acid (TCA) cycle. The TCA cycle is an amphibolic route with anabolic and catabolic reactions. Anaplerotic reactions include the net input of amino acids in the cycle and the generation of oxaloacetate from pyruvate through pyruvate carboxylase. Among the main products of the TCA cycle are guanosine triphosphate (GTP), dinucleotide nicotinamide molecules (NADH), and dinucleotide adenine flavine molecule (FADH_2_); the latter are electron donors to feed the electron transport system (ETS). PDC: pyruvate dehydrogenase complex, ACSS: acetyl CoA synthetase, ACLY: ATP-citrate lyase, Irg1: the immune responsive gene 1 protein, CS: citrate synthase, IDH: isocitrate dehydrogenase, AKG DH: alpha-ketoglutarate dehydrogenase, SS: succinyl CoA synthetase, SDH: succinate dehydrogenase, FH: fumarate hydratase, MDH: malate dehydrogenase, NAD^+^: nicotinamide adenine dinucleotide (oxidized form), FAD: flavine adenine dinucleotide (oxidized form), GDP: guanosine diphosphate. Created with Biorender.com.

The main recognized products of the TCA cycle are one molecule of GTP, three molecules of NADH, and one molecule of FADH_2_; these last are the electron donors to feed the ETS (Figure 1). However, each of the TCA cycle intermediates can also exert different functions and may be involved in kidney diseases.

## 3. Acetyl-CoA

Although acetyl-CoA is not inside the TCA cycle, it is a highly relevant molecule as it is required in the first step, which reacts with OAA to give rise to citrate; interestingly, citrate can be shuttled out from the mitochondrial matrix to the cytosol, where it can be re-converted to acetyl-CoA and OAA.

Acetyl-CoA is generated from different sources, such as the mentioned citrate, which is exported from mitochondria to the cytosol through the SLC25A1 transporter [10], in this compartment, by the action of the ATP-citrate lyase (ACLY) generates OAA and acetyl-CoA; from pyruvate decarboxylation by the action of pyruvate dehydrogenase complex (PDC); from acetate by the action of acetyl CoA synthetase (ACSS); and acetoacetyl-CoA by the action of thiolase. Acetyl-CoA serves as a substrate for fatty acids synthesis and protein acetylation.

Regarding acetyl-CoA involvement in lipid metabolism, fatty acid metabolism impairment has been reported in kidney diseases [11]. In diabetic nephropathy, although acetyl-CoA levels have not been directly reported, the increase in acyl-CoA levels, acetyl-CoA carboxylase, and fatty acid synthase suggest its utilization for fatty acid synthesis, whereas fatty acid oxidation is blocked [12]. The above leads to lipid accumulation and lipotoxicity; similar results were found in animal models of kidney fibrosis induced by folic acid [13], ischemia/reperfusion (I/R) [14], and unilateral ureteral obstruction (UUO) [15]. In renal cell carcinoma (RCC), fatty acid metabolism also seems to be impaired due to altered expression enzymes involved in fatty acid metabolism [16] and intracellular lipid accumulation driven by hypoxia [17]. Interestingly metabolic stress such as hypoxia and low nutrient availability has been reported to induce ACSS expression in cells from breast cancer [18]. This finding suggests that hypoxic alterations that are common in acute kidney injury (AKI) [18], chronic kidney disease (CKD) [19], and renal carcinoma [20], could also contribute to ACSS expression and activation to promote acetyl-CoA synthesis for its further utilization for de novo fatty acid synthesis in these pathologies. In summary, the evidence indicates an impairment in lipid metabolism, with increased acetyl-CoA utilization for fatty acid synthesis and decreased fatty acid oxidation in some kidney diseases.

Acetyl-CoA also serves as a substrate for protein acetylation, a post-translational modification (PTM); if acetylation occurs on lysine (K) residues of histones, this serves as an epigenetic modification for gene expression regulation. In general, histone acetylation by histone acetyltransferases (HAT) elicits genetic expression by inducing chromatin relaxation and favoring the binding of nuclear factors, whereas the absence of histone acetylation could act as a repressive mark [21].

In CKD development by high salt diet and unilateral ureteral obstruction (OUU) in rats, global acetyl-CoA levels are decreased [22,23].

In a transcriptomic analysis of kidney fibroblasts/myofibroblast derived from OUU, transforming growth factor-beta (TGF-β) signaling induces a metabolic reprogramming with reduced TCA cycle-related enzymes, including PDC [23,24], resulting in reduced acetyl-CoA synthesis and levels; moreover, the global acetylation is reduced, and as a consequence, histone acetylation also is decreased [23,24]. Interestingly, the restoration of acetyl-CoA levels partially reverses the induction of fibrotic markers alpha-smooth actin (α-SMA) and collagen [23]. Histone acetylation is a dynamic process since global acetylation of kidney fibroblasts/myofibroblast derived from OUU is decreased, impacting mainly on Histone 3 (H3) K4, K14, and K23 residues; in contrast to K18, and K27 residues that remain acetylated [24]. In the case of H3K9, acetylation is controversial; Smith et al. (2019) found a decrease in UUO rats [24], whereas Hewitson et al. (2017) found an increase in UUO [25] as occurs in diabetic nephropathy [26] in mice. These findings could be explained by the time of disease progression and the animal model. Regarding the above mention, mesangial cells in hyperglycemic conditions induce fibrotic gene expression in an ACLY-dependent manner, involving the increase of H3K9 acetylation [27]. Similarly, in kidney damage induced by obesity, ACLY, the enzyme that generates acetyl-CoA from citrate is increased, and its inhibition in vitro reduces histone acetylation and the expression of lipogenic and pro-fibrotic genes [28]. However, this effect could be in response to nutrient excess.

In AKI induced by I/R, a metabolic reprogramming also has been reported with a glycolytic shift and PDC inhibition [29], indicating a decreased activity of this enzyme complex and ergo a reduction in acetyl-CoA synthesis; moreover, in AKI induced by cisplatin, H3K27 acetylation is reduced, and the restoration of its acetylation levels decreases kidney damage [30,31]. Hence, in CKD and AKI, there appears to be a decrease in acetyl-CoA levels, which in turn impacts histone acetylation.

Contrary, in RCC, acetyl CoA synthetase 2 (ACSS2), the enzyme that produces acetyl CoA from acetate shows increased levels, and in vitro, the expression of this enzyme promotes cell migration [32,33]. In human renal cell adenocarcinoma cell lines, the expression of the snail family transcriptional repressor 1 (SNAI1) is promoted by ACSS2 [34]. SNAI1 is a transcriptional repressor involved in the progression of many types of cancer [35], including RCC [36]. Interestingly, although the global levels of H3 acetylation seems to be decreased in RCC [37], the acetylation of H3K27 seems to be necessary for SNAI1 expression, particularly under hypoglycemic conditions [34].

**Figure 2:**
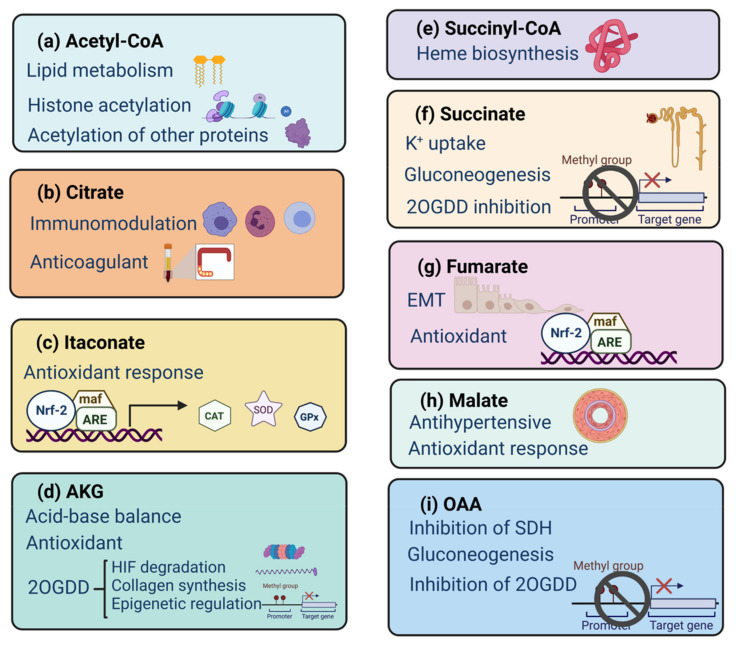
Involvement of TCA cycle metabolites in kidney functions. The metabolites of the TCA cycle and acetyl-CoA participate as signal molecules to promote several critical cellular functions such as epigenetic modifications, redox regulation, hypoxic response, and immunity. Nrf-2: nuclear factor erythroid 2–related factor 2, ARE: antioxidant response element, maf: musculoaponeurotic fibrosarcoma protein, CAT: catalase, SOD: superoxide dismutase, GPx: glutathione peroxidase, 2OGDD: named 2-oxoglutarate dependent dioxygenases, HIF: hypoxia inducible factor, EMT: epithelial-mesenchymal transition, SDH: succinate dehydrogenase. Created with Biorender.com.

Based on the above data, in kidney diseases, there is a complex regulation of acetyl-CoA synthesis and its utilization as a substrate for histone acetylation, which in turn impacts on epigenetic regulation of the gene expression (Figure 2a).

## 4. Citrate

Citrate can be obtained by uptake from dietary sources, and in mitochondria, as a result of the condensation of acetyl-CoA with OAA by the citrate CS; cytoplasmic citrate can be derived from mitochondrial export through the SLC25A1 transporter, also named citrate–malate exchanger (CIC) [10].

Physiologically, an excess of citrate also serves as a control point of glycolysis by inhibiting phosphofructokinase (PFK) [38] and PDC [39], limiting the fructose-1,6-bisphosphate and acetyl-CoA synthesis, respectively [38].

In a model of CKD by UUO or by diabetic nephropathy, urinary excretion of citrate is decreased [40,41], as occurs in patients with CKD [42,43,44]; in contrast, in plasma [45] and kidney tissue [46], citrate levels are increased in UUO. In addition, in cisplatin-induced CKD, CS activity is increased. In contrast, aconitase activity is reduced [47], suggesting that produced citrate is not converted to isocitrate. In non-diabetic CKD patients, the expressions of aconitase 1 and aconitase 2 are reduced; and in urine and blood, the levels of isocitrate are also decreased [42]. In addition, it is known that CS is stimulated by aldosterone [48], a hormone increased in CKD [49], suggesting that in CKD, aldosterone promotes an excess of citrate synthesis. This suggests that produced citrate (probably in excess) is not converted into isocitrate, and its retention results in the reduced urinary excretion [42], as has been demonstrated in animal models of UUO-induced CKD and I/R-induced AKI, in which kidney tissue reveals an accumulation of this metabolite [46,50].

Clinically, urinary low citrate excretion is proposed as a marker of acid retention and reduced glomerular filtration in patients with CKD [43], and plasma citrate levels correlate negatively with estimated glomerular filtration rate (eGFR) [51].

However, in diabetic nephropathy, urinary citrate excretion is controversial due to in humans being decreased [37], whereas in mice it is increased [43], although this may be the result of other metabolic disorders involved in diabetes.

Administration of citrate has been used to manage kidney diseases such as kidney stones [52], AKI, and CKD [53,54,55]. In kidney injury by kidney stones, citrate binds to calcium, preventing its binding to oxalate or calcium phosphate and the consequent reduction of stone formation; however, its effectiveness is still controversial [56].

Citrate administration in AKI and CKD is used as an anticoagulant during renal replacement therapy [53,54,55]. Moreover, in a model of AKI by I/R, citrate administration reduces plasma creatinine levels, lactate dehydrogenase activity and partially restores ATP content in tissue, reflecting improvement in kidney function [57]. Interestingly, citrate has also been associated with immunomodulatory effects. In AKI patients with continuous venovenous hemofiltration therapy, citrate administration reduces myeloperoxidase and interleukin 8 (IL-8) plasma levels [58]; in a model of CKD induced by adenine in rats, the administration of citrate reduces the production of pro-inflammatory cytokines interleukin 6 (IL-6) and interleukin 17 (IL-17), whereas it increases the anti-inflammatory cytokines interleukin 10 (IL-10) and TGF-β [59].

The immunomodulatory effects of citrate have also been reported in other cells types such as monocytes and macrophages. In these cells, ROS and pro-inflammatory cytokines were reduced in response to lipopolysaccharide (LPS) [60,61]; however, this effect could be dependent on citrate concentration [61].

In RCC, citrate levels are enriched [62], and its immunosuppressive effects could be related to the tumor progression; however, there is still no evidence of this effect. However, in RCC, citrate is re-converted to acetyl-CoA by ACLY, which in turn serves as the substrate for protein acetylation and fatty acid synthesis; as mentioned above, RCC also has elevated levels of ACLY. Interestingly, it is silencing, avoiding citrate-derived acetyl-CoA, promoting apoptosis, and reducing proliferative and migration rates in RCC cells [63].

Citrate involvement in kidney diseases includes immunomodulatory effects, regulating acetyl-CoA synthesis, and even being used in their therapeutic management (Figure 2b).

## 5. Isocitrate/Itaconate

Aconitase is the enzyme responsible for the conversion of citrate to cis-aconitate and later to isocitrate.

Aconitase is an iron–sulfur-containing dehydratase; its activity is sensitive to oxidation [64]. In kidney diseases, it is well-known that oxidative stress is a hallmark [65], hence suggesting that aconitase activity is reduced, as reported in CKD induced by cisplatin [47] and 5/6 nephrectomy [66]; as well in AKI induced by maleate [67] and I/R [68].

In addition, as kidney function declines in nephrectomy-induced CKD [61], aconitase activity also decreases [69].

In non-diabetic CKD, isocitrate urinary excretion, aconitase 1 (mitochondrial aconitase), and 2 (cytosolic aconitase) expression are reduced in kidney tissue [42].

At present, there is no evidence of isocitrate as a signal molecule, and its synthesis seems to be decreased in kidney diseases. On the other hand, itaconate is another intermediate of the TCA cycle derived from the decarboxylation of cis-aconitate by the immune responsive gene 1 protein (Irg1). Interestingly, itaconate seems to have immunomodulatory effects [70].

In the I/R model, Irg1 levels increase after 12 h, with a peak at 24 h; the induction of this enzyme on the different cell types depends on the stimulus. For example, renal cells respond to H_2_O_2_, increasing Irg1 levels, whereas macrophages respond mainly to pro-inflammatory stimulus, such as cytokines and cell lysates, and lesser extent to H_2_O_2_ [71]. Itaconate has protective effects since Irg1 knock-out mice exacerbate inflammatory response and reduced survival percentage induced by I/R [71]. Due to this immunomodulatory effect, this metabolite has been used for reducing damage in kidney tissue and cells.

The administration of 4-octyl itaconate (OI), a derivate of itaconate with higher fat solubility, by tail vein injection, reduces fibrotic kidney damage induced in UUO or by adenine administration in rats. This effect was partly through the reduction of the canonical signaling of TGF-β pathway and by recovering antioxidant enzyme expression in adenine-induced kidney damage or decreasing inflammatory response by reducing nuclear factor kappa B (NF-κB) activation in UUO [72]. In vitro, OI treatment also reduces fibrotic markers fibronectin, plasminogen activator inhibitor 1 (PAI-1), and α-SMA, decreases phosphorylation of p65 subunit of NF-κB; whereas stimulates antioxidant response through the increase of the nuclear factor erythroid 2-related factor (Nrf2) and reducing ROS levels in kidney epithelial cells HK-2 stimulated with TGF-β [72].

Dimethyl itaconate (DMI), another derivate of itaconate, has also been demonstrated to have a renal protective effect. The treatment of neonatal renal cells with DMI and exposure to hypoxia/reoxygenation (H/R) reduces cell death; in addition, the antioxidant response is activated due to the increase in Nrf2 nuclear translocation [71]. A similar result was demonstrated in macrophages exposed to H/R, in which DMI reduces inflammatory response by decreasing tumor necrosis factor-alpha (TNF-α) and interleukin 1-beta (IL-1β) production through reducing mitogen-activated protein kinase (MAPK) and NF-κB activation; this effect was in part due to the induction of antioxidant response mediated by Nrf2 stimulation [71].

The well-reported mechanism of action of itaconate is through its binding to Kelch-like ECH associated protein 1 (KEAP1), a negative regulator of Nrf2, the interaction of itaconate with KEAP1, elicits the dissociation of this last one from Nrf2, inducing the nuclear translocation of Nrf2 to promote antioxidant gene expression [73]. Interestingly, itaconate also inhibits SDH activity, which results in succinate accumulation and the inhibition of fumarate formation [74]; both mechanisms are demonstrated in monocytes/macrophages. Itaconate has also demonstrated antimicrobial functions, blocking the glyoxylate cycle in *Mycobacterium avium* and *Mycobacterium tuberculosis* [75] (Figure 2c).

Although itaconate immunomodulatory effects are an emerging and growing field, there is little evidence of its levels and action mechanisms on kidney diseases.

## 6. Alpha-Ketoglutarate

The decarboxylation of isocitrate by the IDH gives rise to 2-oxoglutarate, which is also named AKG, which can also be derived from glutaminolysis [76] and even can be produced by microorganisms [77].

At present, there is little evidence related to AKG alterations in kidney diseases, such as increased urinary excretion in diabetic nephropathy in humans [44] and mice [78]; however other report decreased urinary levels in humans and mice [42,79] and reduction of blood serum AKG concentration in diabetic nephropathy in mice [41]. On the other hand, in RCC, contrary results were found in tissue with an elevated concentration in mice [80] compared to low levels reported in human samples [81].

Even though the information related to AKG levels in kidney disease is limited and confusing, this molecule and the enzymes related to its metabolism seem to be of great relevance in different kidney conditions.

As mentioned above, IDH catalyzes the conversion of isocitrate to AKG. IDH1 is expressed in cytosol and peroxisomes, whereas IDH2 and IDH3 are expressed in mitochondria. IDH1 and IDH2 are nicotinamide adenine dinucleotide phosphate (NADP)-dependent, and each one functions as homodimers; whereas IDH3 is nicotinamide adenine dinucleotide (NAD)-dependent and is composed by three different subunits; thus resulting in the production of the reduced form of NADP (NADPH) by IDH1/2, or NADH by IDH3 in addition to the AKG synthesis [82]. It is well known that NADPH is a substrate for antioxidant defense, used for glutathione regeneration and thioredoxin activity [83,84,85]. In addition, the administration of AKG also has been reported to function directly as an antioxidant [86,87,88], as mentioned below.

In acute kidney injury by cisplatin, IDH2 levels are decreased; moreover, IDH1/2 activities are reduced, but with no IDH3 [89]; in a similar way, in I/R-induced AKI it has been reported that IDH1/2 are reduced, as well their function [83,90]; on the other hand in UUO-induced CKD, IDH2 levels also are reduced, and in diabetic nephropathy, also IDH2 activity is diminished [91]. Thus, demonstrating that IDH2 activity reduction is a common characteristic in all of these pathologies. Besides, genetic deletion of IDH2 exacerbates renal damage by increasing oxidative stress and leukocyte infiltration in I/R and cisplatin-induced AKI and UUO-induced CKD models [83,89,92], reflecting an antioxidant protective effect of this enzyme additional to its function of AKG synthesis. Additionally, an interesting finding is that in diabetic nephropathy, IDH2 deficiency also increases the expression of renin, angiotensin II type 1 receptor, angiotensinogen, and angiotensin-converting enzyme in renal tissue, as well renin and angiotensin II levels in plasma, promoting hypertension derived from oxidative stress [91].

In non-diabetic CKD patients, IDH3 expression is decreased [42], which suggests an impairment in AKG and NADH+ synthesis, resulting in low levels of electron donors for the ETS. On the other hand, in RCC, low expression of IDH1 has been associated with a poor prognosis [93]. Independent of which IDH catalyzes the synthesis of AKG, this metabolite exerts different functions in the kidney.

A new physiological function of AKG participating in the acid–base balance in the kidney has been reported. At the extracellular level, AKG can be recognized by its receptor 2-oxoglutarate receptor 1 (OXGR1), which is expressed in cells of the connecting tubule and cortical collecting tubule; once activated, this receptor acts in conjunction with pendrin, regulating the HCO_3_^−^ excretion and NaCl reabsorption [94].

AKG also has been reported to function as a non-enzymatic antioxidant, scavenging H_2_O_2_ and enhancing the activity of other antioxidant molecules in liver damage induced by ethanol or acetaminophen, respectively [86,87]. Thus, in a model of hyperammonemia, liver and kidney damage were reduced by the oral administration of AKG, restoring the antioxidant status in both organs [88]. In vitro, kidney proximal tubules under hypoxic condition show mitochondrial alterations and decreased ATP levels; however, the use of AKG in combination with aspartate reduce mitochondrial structural alterations and partially restores ATP levels by replenishing TCA cycle [95]. In a similar approximation in AKI induced by I/R, the treatment with AKG plus malate did not demonstrate protective effects in the kidney, even as a deleterious effect, mean arterial blood pressure (MAP) and heart rate were decreased [96]. Although the AKG was not promising for AKI treatment, hypertension in CKD is a concomitant alteration [97], opening a new exciting research field of the effect of AKG on CKD progression.

In addition, AKG also participates in the function of a superfamily of enzymes named 2-oxoglutarate dependent dioxygenases (2OGDD). The reaction catalyzed by 2OGDD is the hydroxylation of the substrate, requiring as co-substrates O_2_, Fe^2+^, and AKG. Prolyl hydroxylases (PHD), histone demethylases (HDM), nucleic acid oxygenases, and fatty acid oxygenases are some of the 2OGDD [98]. Some structural analogs of AKG, such as pyruvate, citrate, isocitrate, succinate, fumarate, malate, OAA, R-2-hydroxyglutarate (R2HG), and L-2-hydroxyglutarate (L2HG), act as 2OGDD inhibitors [98].

PHD function hydroxylating proline residues of several proteins, such as the hypoxia-inducible factor (HIF) promoting its proteasomal degradation; and collagen, inducing its structural conformation. In I/-R-induced AKI, the pre-treatment, but no post-ischemic damage, with a PHD inhibitor (PHI), GSK1002083A, reduces the fibrotic lesions and maintains kidney function [99]. Similarly, in AKI induced by cisplatin and folic acid, the pre-treatment with other PHI, FG-4592, also decreases kidney damage, reducing the inflammatory and fibrotic responses [100,101]; however, in UUO-induced CKD, the use of PHI does not affect fibrotic or inflammatory markers [102], demonstrating that inhibition of PHD is effective only in acute damage. The action mechanism of PHI is through avoiding HIF proteasomal degradation as an acute protective response [99,100,101]; however, although it was not demonstrated, possible inhibition of collagen synthesis could also contribute to PHI benefits in kidney diseases. Since HIF is necessary for erythropoiesis induction, PHI roxadustat and GSK1278863 also has been proposed for anemia treatment in CKD, demonstrating the increase in erythropoietin and hematocrit levels in animal models [103,104] and even in patients [105]; hence in CKD and AKI, there is an increase in PHD activity, and their inhibition ameliorates damage associated with kidney dysfunction.

Another 2OGDD enzymes involved in kidney diseases is the ten-eleven translocation methyl-cytosine dioxygenase (TET)1-3, which catalyzes the conversion of 5-methyl cytosine (5mC) to 5-hydroxymethyl cytosine (5hmC), an epigenetic mark on DNA associated with active transcription [106]. In tissue derived from CKD patients, TET1-3 levels are increased; however, their activity is decreased [107], indicating an imbalance in 5mC/5hmC. TET2 low activity is associated with worsen acute kidney damage induced by I/R [108,109] and cisplatin [110]; whereas TET3 low activity is associated with chronic kidney damage induced by UUO [111,112]; moreover, restoration of TET function ameliorates kidney injury [107,110,112] by the hydroxymethylation of different genes such as Klotho [107] and ras protein activator like 1 (RASAL) [111] promoters, which codify for renoprotective and anti-proliferative proteins, respectively. In addition, in UUO and nephrectomy models of kidney damage, the inhibition of the histone demethylase Jumonji domain containing-3 (JMJD3), another 2OGGD, worsen fibrotic lesions through enhancing TGF-β signaling [113]. Thus, in kidney injury seems that removing DNA and histone methylation by 2OGDD demethylases has protective effects.

It is crucial to notice that the 2OGDDs function depends on their expression, the availability of co-substrates AKG, O_2_, and Fe^2+^, and the presence of structural inhibitors. For example, in RCC, despite the increase of the 2OGDD histone and DNA demethylases, there is a reduction in 5hmC levels. This finding is probably secondary to the low availability of AKG and the presence of its structural analog L2HG [114].

In summary, the AKG, the enzymes involved in its synthesis, and the enzymes that require it for their function seem to be highly relevant in kidney diseases development. Here we mention only a few examples of 2OGDD; however, these enzymes are involved in a great variety of biological functions (Figure 2d).

## 7. Succinyl-CoA

Succinyl-CoA is derived from AKG by the action of the AKG dehydrogenase, also named 2-oxoglutarate dehydrogenase (2OGDH); succinyl-CoA can also be derived from succinate by the action of the succinyl-CoA synthetase.

In addition to its role in the TCA cycle, succinyl-CoA and glycine participate in the biosynthetic heme pathway, necessary for different kidney hemoproteins synthesis.

2OGDH is decreased in CKD induced by high-salt diet and aristocholic acid administration [22,115], as has been reported in non-diabetic CKD patients [42]. On the other hand, in RCC 2OGDH lower expression was associated with poor outcome [116]. Together, this indicates a decreased synthesis of succinyl-CoA in some progressive kidney diseases.

P450 cytochromes (CPY) are hemoproteins that have been associated with kidney dysfunction. In end-stage renal failure, a decrease in CYP1A2, CYP2C9, CYP2C19, and CYP3A4 expression in blood samples has been reported [117]; these CPYs are associated with drug excretion. On the other hand, CPY24, an enzyme that regulates vitamin D levels, seems to be increased in adenine-induced CKD [118]. However, there is no evidence that succinyl-CoA levels in kidney diseases drive altered expression or synthesis of CPY.

Other hemoproteins include catalase, nitric oxide synthase, and prostaglandin synthase; however, in kidney diseases, these enzymes are evaluated by their function rather than their synthesis derived by the succinyl-dependent biosynthetic heme pathway (Figure 2e).

Currently, there is little evidence related to the direct role of succinyl-CoA in kidney pathologies.

## 8. Succinate

Succinate is derived from succinyl-CoA by the reaction of succinyl CoA synthetase. In high-diet-salt-induced CKD, succinate levels decrease in tissue [22], as has been reported in non-diabetic patients with CKD with decreased levels in kidney biopsies and low urinary excretion [42]. In diabetic nephropathy in rodents, succinate levels are increased in urine [41,78,119], whereas in kidney tissue are reported both, decreased [119] and increase levels [120]. In contrast, in UUO, succinate levels increase in plasma and kidney tissue [45,46], as happens in I/R-induced AKI [50,121] and polycystic kidney disease [122]. In RCC also increased succinate levels has been found [62].

Hence, it seems that there is a dynamic regulation of succinate levels and its excretion depending on kidney damage injury.

An exciting finding from almost two decades ago was the discovering of succinate receptor 1 (SUCNR1) in the kidney, which is found mainly in the proximal tubules [123].

It is known that in the proximal tubule, succinate stimulates gluconeogenesis [124] and induces membrane hyperpolarization by increasing K^+^ uptake [125], although it is uncertain if these functions depend on SUCNR1. However, a well-documented function of succinate/SUCNR1 signaling is the stimulation of arachidonic acid, prostaglandin E2, and prostaglandin I2 release, which in turn stimulates renin release [120,123,126,127]. Moreover, in diabetic nephropathy in mice, hyperglycemia induces renin release through SUCNR1 [120]. In CKD, there are alterations in the renin/angiotensin/aldosterone axis; however, currently is unknown if succinate and its receptor are participating.

Another reported function of succinate is its inhibitory effect on 2OGDD mentioned above, particularly inhibiting PHD and indirectly stabilizing HIF [128]. Regarding above mentioned, succinate has also been described as a pro-inflammatory signal promoting the expression IL-1β via HIF activation in macrophages [129], opening a new panorama of the participation of this metabolite during the inflammatory response in kidney diseases (Figure 2f).

## 9. Fumarate

SDH catalyzes the reaction that transforms succinate to fumarate; this reaction also can be in the direction of fumarate to succinate. Interestingly, SDH also is named complex II and is part of the ETS.

This metabolite also can be derived from arginosuccinate as a part of the urea cycle. Fumarate increased levels in the plasma of diabetic and non-diabetic CKD in mice [41,42], whereas increased urinary excretion and levels in the renal cortex in diabetic mice [78] are reported. In contrast, in kidney damage induced by cisplatin, decreased urinary excretion has been found [130], similarly in injury induced by I/R and adenine, decreased tissue fumarate levels [50,131] has been reported. In RCC, fumarate also appears to be reduced in tissue [62]. As happens with other TCA cycle metabolites, its regulation seems to be dynamic.

Besides, SDH activity is reduced in CKD models induced by potassium dichromate [132], sulfasalazine [133], cisplatin [134], and UUO [135]. In addition, the exposure of proximal tubular epithelial cells to uremic toxins decreases SDH activity [136], suggesting that fumarate is not synthesized and the ETS is not working fully. In comparison, during acute injury by I/R, SDH blockade with malonate has protective effects in the kidney [137]; however, also reduction in its activity has been reported in this model [138]; hence, deeper studies are necessary to understand the molecular mechanism of the SDH under the specific condition of renal damage.

In addition, fumarate, as succinate, is a 2-OGDD inhibitor and has exceptional attention in a subtype of RCC (FH-deficient RCC), in which an FH mutation avoids the conversion of fumarate into malate, leading to an excessive fumarate accumulation [139]. Fumarate accumulation has been demonstrated to induce epithelial-mesenchymal transition (EMT) through epigenetic regulation inhibiting TET demethylase. The above, finally provides phenotypic mesenchymal characteristics and migratory capacities to the cells, thus is highly relevant in the progression of RCC [140]. In other kidney disorders, such as CKD-induced fibrosis, EMT is a phenomenon also observed [141,142] in which the fumarate role has not been elucidated.

In addition, fumarate seems to have protective effects in the kidney, as demonstrated in kidney damage induced by ciclosporin, cisplatin, folic acid, and I/R [143,144,145], in which dimethyl fumarate administration reduce kidney damage by enhancing the antioxidant response driven by Nrf2 (Figure 2g). Moreover, dimethyl fumarate is already approved by the food and drug administration (FDA) as an immunomodulatory drug for the therapeutic management of multiple sclerosis [146].

## 10. Malate

Malate is raised from fumarate by FH action and from pyruvate by the action of the malic enzyme.

There is little evidence of malate alterations in kidney diseases, such as increased levels in serum and urine in diabetic nephropathy in mice [41,78] and reduced levels in kidney tissue from RCC and I/R injury [50,62]. In fact, the reduction of FH activity has been proposed as a biomarker of acute kidney injury [147]. The silencing of FH in HK-2 renal epithelial cells increases fumarate levels, whereas it decreases malate levels as expected; interestingly, it also reduces nitric oxide levels and the activity of nitric oxide synthase (NOS) [148], which is known to induce vascular relaxation. In a model of hypertension in rats, malate administration increased NOS levels and activity, and alleviated hypertension, reducing the MAP [148]. Similar results were obtained in a model of I/R in which malate administration plus AKG causes hypotension reducing the MAP [96].

In addition, malate synthesis by the malic enzyme is highly relevant due to the formation of NADPH for glutathione and thioredoxin antioxidant activities [149]. Related to the above, in kidney damage the cisplatin malic enzyme increases its activity [145], probably as a reparative mechanism (Figure 2h).

## 11. Oxaloacetate

OAA can be synthesized from malate by the MDH. It can also be derived from pyruvate catalyzed by the pyruvate decarboxylase, or aspartate by the glutamic oxaloacetate transaminase (GOT). OAAs can be condensed with acetyl-CoA to start the cycle again and also can be used for gluconeogenesis.

Currently, there is no information related to OAA levels in kidney diseases, probably by the difficulties in its measurement [150]. However, in kidney injury induced by toxic compounds potassium dichromate [151], gentamicin [152], melamine/cyanuric acid, and in diabetic nephropathy [153], MDH activity reduction has been reported; suggesting a decrease in OAA synthesis. Moreover, GOT serum levels in CKD patients are reduced and correlated with advanced stages of the disease [154].

Contrary, in RCC, MDH and GOT expression are increased [155], suggesting an increase in OAA synthesis. Furthermore, OAA inhibits SDH [156], thus impacting ETS activity and promoting succinate accumulation, which can inhibit 2OGDD as mentioned above (Figure 2i).

## 12. Clinical Significance of TCA Metabolites

Clinically, kidney function is evaluated by indirect measurement of glomerular filtration by serum creatinine levels, albuminuria, proteinuria, and eGFR. Recently, the use of mass spectrometry (MS) as a tool with proteomics [157], peptidomics [158], and metabolomics [159] approaches to the discovery of new biomarkers in urine and serum, has increased, showing a large number of molecules with potential use in the clinic. Some examples of molecules identified by mass spectrometry currently useful as biomarkers in clinics include cystatin C [160,161], neutrophil gelatinase-associated lipocalin (NGAL), and kidney injury molecule 1 (KIM1) [162,163,164,165]. Hence, the use of new biomarkers in conjunction with the classical method of kidney function evaluation could be helpful in a more accurate diagnosis or prognosis of different kidney diseases.

Due to the involvement of TCA cycle metabolites in kidney physiology and pathophysiology, identifying these in biofluids, such as serum and urine by metabolomics, could give insights into their use as potential biomarkers in different kidney diseases.

**Acetyl-CoA.** Currently, acetyl-CoA has not been identified as a biomarker in kidney diseases, probably by its multiple sources and its implication in diverse biochemical pathways. However, as mentioned above, one of its functions is in the fatty acid metabolism, which seems to be impaired in kidney diseases [11]. Carnitine can react with acetyl-CoA to form acetyl-carnitine during fatty acid metabolism by the carnitine acetyltransferase (CAT).

In CKD, serum levels of acetyl-carnitine increase along with disease progression, whereas in urine are decreased; even more, serum acetyl-carnitine shows a negative correlation with eGRF [166,167]. In AKI patients, serum levels of acetyl-carnitine levels also are increased [168]. In biopsies of renal cell carcinoma, acetyl-carnitine is increased; moreover, there are differences between clear cell, papillary, and chromophobe subtypes, with a more noticeable increase in clear cell RCC subtype [169].

Currently, acetyl-carnitine has been proposed as a biomarker for hepatocellular carcinoma, in which it is increased [170,171]; and in major depressive disorder, in which levels are decreased in serum. In kidney diseases, the use of this metabolite and the eGFR could help evaluate kidney function. However, more in-depth studies are necessary to determine its utility in discriminating against different kidney diseases.

**Citrate.** As mentioned above, urinary excretion of citrate is decreased in patients with CKD [42,43,44]. Clinically, urinary low citrate excretion is proposed as a marker of acid retention and reduced glomerular filtration in patients with CKD [43]. The meaning of plasma citrate is not clear enough since both negative and positive correlations with estimated glomerular filtration rate (eGFR) have been proposed [51,172]; in addition, the ratio of myo-inositol:citrate in urine seems to predict active renal vasculitis [173]. Similarly, in AKI pediatric patients, urinary citrate levels were found to be reduced [174]. In RCC, citrate levels decreased in urine [175], but these are enriched in tissue [62]. Hence, reduced citrate levels in urine seem to be a promising biomarker of altered kidney function.

**Isocitrate.** There is scarce information related to isocitrate alteration in serum or urine levels in kidney diseases. This metabolite and its derivate cis-aconitate are decreased in the urine of CKD patients [42]; additionally, plasma isocitrate correlates negatively with eGFR [51], being a possible predictor of disease progression. On the other hand, in RCC, low expression of IDH1, the enzyme responsible for isocitrate conversion to AKG, has been associated with a poor prognosis [93]. More in-depth studies are necessary to understand the clinical significance of this metabolite.

**AKG.** In CKD patients, decreased urinary levels of AKG have been reported [42], whereas there are no differences in AKI patients [176]. In RCC, AKG urinary excretion is increased [177], whereas, in biopsies, reduced levels have been reported; even more, tissue levels of this metabolite could be helpful in the prognosis of this neoplasia [81].

**Succinate.** In CKD patients, decreased urinary levels of succinate have been reported [42], and plasma succinate correlates negatively with eGFR [51]. In RCC, succinate levels are increased in tissue [62] and decreased in urine [175]. However, there is no information related to alterations of this metabolite in urine or serum from AKI patients. Currently, increased urinary levels of succinate and AKG have been proposed as biomarkers of major depressive disorder [178], opening a new panorama for the use of these metabolites in the clinic.

**Fumarate.** Increased urinary levels of fumarate in CKD patients have been described [44]; also, plasma fumarate correlates positively with eGFR [51] and has been associated with mortality [179]. In RCC, tissue fumarate levels are decreased [62], and no altered levels are reported in AKI patients. However, in an animal model, FH activity in urine and plasma has been proposed as a biomarker of AKI [147].

**Malate.** As fumarate, increased malate levels in the urine of CKD patients have been reported [44], and plasma malate also correlates negatively with eGFR [51]. In RCC, tissue malate levels are decreased [62]. In AKI patients, there are no reported altered levels of this metabolite.

**OAA**. As mentioned above, OAA is difficult to detect by MS; hence, its potential as biomarker is limited. In CKD, GOT serum levels are reduced and correlated with advanced stages of the disease [154]. In RCC tissue, GOT expression is increased. GOT is currently used for clinical evaluation of liver function; its use in conjunction with other parameters in the diagnosis and prognosis of kidney diseases could be helpful in clinics.

A summary of alterations of some TCA cycle metabolites in kidney diseases in humans and their potential as biomarkers is showed in Table 1.

## 13. Concluding Remarks and Future Directions

Mitochondria perform several functions, including metabolic pathways and communicating to the rest of the cell to drive its behavior. The TCA cycle occurs in the mitochondrial matrix, and the metabolites that compose it are dependent on each other; hence the excess or lack of the TCA cycle metabolites are regulated by their release from mitochondria or can be replenished from cytosolic precursors.

As we review, TCA cycle metabolites are involved in several kidney functions in health and disease. Moreover, in kidney diseases, there are alterations in the levels of TCA cycle metabolites and in the enzymes involved in their synthesis, which drive cell fate impacting kidney function.

Knowing the role of these metabolites in kidney diseases is of great relevance to understanding the pathophysiology and for their possible application in future therapeutic options and for clinical use as prognosis/diagnosis biomarkers.

## Figures and Tables

**Table 1:** TCA cycle metabolites alterations in kidney diseases with potential use as biomarkers.

Metabolite	Kidney Disease			References
	CKD	AKI	RCC	
Acetyl-carnitine	Δ serum ∇ urine	Δ serum	Δ tissue	[166,167,168,169]
Citrate	∇ urine	∇ urine	∇ urine	[43,62,174,175]
Isocitrate	∇ urine	-	-	[42]
AKG	∇ urine	-	∇ urine Δ tissue	[42,81,177]
Succinate	∇ urine	-	∇ urine Δ tissue	[42,62,175]
Fumarate	Δ urine	-	∇ tissue	[44,62]
Malate	Δ urine	-	∇ tissue	[44,62]

Summary of tricarboxylic citric acid (TCA) cycle alterations in different kidney diseases in humans. Δ = increased levels, ∇ = decreased levels; CKD, chronic kidney disease; AKI, acute kidney injury; RCC, renal cell carcinoma; AKG, alpha-ketoglutarate.
